# Mold Odor from Wood Treated with Chlorophenols despite Mold Growth That Can Only Be Seen Using a Microscope

**DOI:** 10.3390/microorganisms12020395

**Published:** 2024-02-16

**Authors:** Johnny C. Lorentzen, Olle Ekberg, Maria Alm, Folke Björk, Lars-Erik Harderup, Gunnar Johanson

**Affiliations:** 1 Integrative Toxicology, Institute of Environmental Medicine, Karolinska Institutet, SE-171 77 Stockholm, Sweden; gunnar.johanson@ki.se; 2 Centre for Occupational and Environmental Medicine, Region Stockholm, SE-113 65 Stockholm, Sweden; 3 Division of Building Physics, Lund University, SE-221 00 Lund, Sweden; ekberg.olle@gmail.com (O.E.); lars-erik.harderup@byggtek.lth.se (L.-E.H.); 4 Urban Property Department, SE-402 26 Gothenburg, Sweden; maria.alm@stadsfast.goteborg.se; 5 KTH Royal Institute of Technology, SE-100 44 Stockholm, Sweden; folke.bjork@byv.kth.se

**Keywords:** asthma, allergy, sick building syndrome, pesticides, wood preservatives, confounding, odor, mold, dampness, indoor air

## Abstract

We previously reported that indoor odorous chloroanisoles (CAs) are still being emitted due to microbial methylation of hazardous chlorophenols (CPs) present in legacy wood preservatives. Meanwhile, Swedish researchers reported that this malodor, described since the early 1970s, is caused by hazardous mold. Here, we examined to what extent CP-treated wood contains mold and if mold correlates with perceived odor. We found no studies in PubMed or Web of Science addressing this question. Further, we investigated two schools built in the 1960s with odor originating from crawlspaces. No visible mold was evident in the crawlspaces or on the surfaces of treated wood samples. Using a microscope, varying amounts of mold growth were detected on the samples, all containing both CP(s) and CA(s). Some samples smelled, and the odor correlated with the amount of mold growth. We conclude that superficial microscopic mold on treated wood suffices produced the odor. Further, we argue that CPs rather than mold could explain the health effects reported in epidemiological studies that use mold odor as an indicator of hazardous exposure.

## 1. Introduction

The present work addresses a confusion between microorganisms and pesticides that is deeply rooted in indoor air research and is manifested, for example, in the widespread notion that mold odor is an indicator of hazardous mold [1,2]. As is known today [3,4,5,6,7,8,9], a moldy odor can also be an indicator of chlorophenols (CPs), including pentachlorophenol (PCP), which is classified as a group I carcinogen (“Carcinogenic to humans”) by the International Agency for Research on Cancer (IARC) [10], and a persistent organic pollutant (POP) by the Stockholm Convention [11,12]. These organochlorine chemicals were first marketed in the USA, and being broadly biocidal and cheap to produce, they rapidly found perhaps more varied uses than any other pesticide, for example, in construction industries and in homes to protect against insects and mold [13]. Thus, the CPs and their salts were extensively used during building booms after World War II [7]. For decades, the traditional constructional protection against moisture, which could lead to wood rot, was complemented and even partly substituted with chemical protection against wood decay fungi [5,6,7]. Furthermore, we have highlighted that these new building practices created perfect conditions for odor formation due to a peculiar characteristic of CPs, namely that some microorganisms can methylate them to odor potent chloroanisoles (CAs) [5,6,7].

Already in 1966, it was reported in *Science* that sensory defects of eggs and meat were due to chicken being housed together with wood chips containing CAs [14], formed by microbial methylation of CPs in wood preservative residues [15,16,17]. Soon, CAs were also reported in soil [18,19,20], water [21], and fish [22], and they became notorious for deteriorating the quality of a wide range of consumer items at very low concentrations [23,24,25,26,27]. Various products were spoiled by CAs in buildings, caves, or containers with CPs in treated wood constructions, floors, or pallets [28,29,30].

In the 1970s, concerns were raised about the toxicity of CPs, and they were discovered to contain even more toxic contaminants on a mass basis, such as dioxins and furans. The fact that the CPs were promoted by national authorities and industries in concert [5,6,7] may have played a role in the remarkable circumstance that the pesticides and their odorous derivatives were not recognized as research evolved around frequent indoor air problems [31,32]. The exception is Germany [7], where a major public stir related to CPs occurred [33,34,35], a “wood preservative syndrome” evolved [36], and CAs were recognized as a cause of musty malodor [6,7,37,38].

When indoor malodor and coinciding health complaints evolved in Sweden and other countries, the problems were attributed to mold. We have shown that Sweden and the neighboring Nordic countries did use CPs in buildings, but this could only be ascertained by searching through grey literature, newspaper articles, and advertisements written in Nordic languages [6]. In Sweden, the grey literature reveals that since 1974 [39], impregnated wood has continuously been implicated as a source of the indoor nasty odor that still evolves nationwide from legacy preservatives [6]. The nasty odor was key for research on “sick building syndrome” (SBS), allergy, asthma, etc. [6], and it was described in 1987 by Tomas Lindvall, a leading scientist in the international arena [7], as: “*the salient effect on the occupants of many ‘mould buildings’ is the persistent and annoying odour which frequently causes psychosocial problems*” [40]. Yet, at the time, a government agency had already stated that visible mold was hard to find, even when opening structures, in reports that changed the title from “Houses with mold odor” [41] to “Mold in houses” [42].

Since 1999, Swedish building investigators have used analyses of CPs and CAs to explain mold odor [43]. Unfortunately, these and many other relevant circumstances did not enter, or were not mentioned, in the health science domain. Thus, when 50 years of international indoor air research were described in 2017 by Jan Sundell, a former Ph.D. student of Lindvall and a leading scientist in the international arena [7], the toxic CPs and odorous CAs were not even mentioned, whereas research on mold and mold odor was accounted for and reflected upon [32].

**Figure 1:**
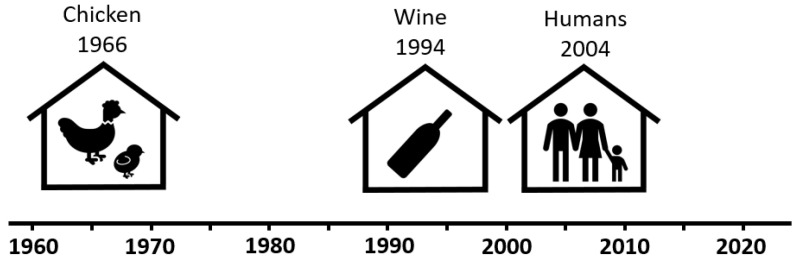
Timeline of scientific reporting of sensory problems caused by indoor CA(s). The timeline is based on references from the poultry, wine, and housing sectors [14,28,29,37]. The timeline will differ slightly if based on other information sources. For example, the German 2004 scientific report on CAs causing musty odors in houses [37] was preceded by a non-scientific document in Swedish from 1999 on CAs causing moldy odors [43].

In our earlier studies, we concluded that the impact of CPs on indoor environments has been confused with mold. This explains why the odorous CAs were only recognized in Germany and Sweden and why this occurred so late in both countries, around 30–40 years after the problematic chemicals were recognized in the housing of chickens (see Figure 1).

Given that Swedish impregnated wood played a previously unrecognized key role in forming the perception of hazardous mold, we aimed, in the present work, to answer the questions (i) to what extent CP-treated wood contains mold and (ii) whether the amount of mold correlates with perceived odor. To address these questions, we performed experimental work in two Swedish schools with odor problems and searched international scientific literature for relevant data.

## 2. Materials and Methods

### 2.1. Litterature Search on Relations between CPs, Cas, and Mold

PubMed and Web of Science were searched using the following search string:

“(mold OR mould OR mildew) AND (chlorophenol* OR monochlorophenol* OR dichlorophenol* OR trichlorophenol* OR tetrachlorophenol* OR pentachlorophenol* OR chloroanisole* OR monochloroanisole* OR dichloroanisole* OR trichloroanisole* OR tetrachloroanisole* OR pentachloroanisole*)”. In addition to the chemical names, all relevant CAS numbers (see File S1, Table S1) were included in the search. The searches were performed on 16 October 2023.

### 2.2. Investigation of Two Swedish Schools with Odor Problems

#### 2.2.1. Object Selection

Two elementary schools, both located in Gothenburg, Sweden, were investigated. They were selected in collaboration with the City of Gothenburg’s Urban Property Department because of the following characteristics: being constructed in the 1960s–70s; documented problems with indoor malodor; remedial actions targeting odor from crawlspaces due to CPs/CAs; easy access to crawlspaces for inspection and sampling of wood.

#### 2.2.2. Crawlspace Inspection and Sampling of Treated Wood

Crawlspaces were inspected and sampled by Olle Ekberg, who previously worked as a building investigator. Various locations were sampled using a chisel that was cleaned with denatured alcohol between each sampling. The collected samples, 15 in total, were 3 cm wide and 5–10 cm long wooden slivers carved from sill plates and crawl space ceilings without touching the surfaces, then wrapped in aluminum foil and put in marked polyethylene zip-lock bags. The damage caused by sampling was superficial, did not affect the structure, and was approved by the building owner.

#### 2.2.3. Analysis of Mold

Assessment of visible mold was made by looking vertically down at a fixed distance of 30 cm from the sample lit from four directions. Photographs were taken under the same circumstances. Microscopic mold on wood samples was assessed by an experienced microscopist at Lund University using a mold index scale (MI) ranging from 0 to 4 [44]. It was performed using an Olympus SZX7 stereo microscope (Shinjuku, Tokyo, Japan) at 40× magnification and low-angle light to detect fungal structures: hyaline (transparent) or dematiaceous (brown-colored) hyphae and conidiophores (spore-producing hyphae) [44]. The MI scale is as follows: 0 = no mold growth; 1 = initial growth, with one or a few hyphae and no conidiophores; 2 = sparse but clearly established growth, often conidiophores are beginning to develop; 3 = patchy, heavy growth with many well-developed conidiophores; and 4 = heavy growth over more or less the entire surface [44].

#### 2.2.4. Evaluation of Odor

The odor from samples was evaluated in odor-neutral laboratory rooms in Lund and Uppsala. Each sample was removed from the zip-lock bag, unwrapped from aluminum foil, and then held close to the nose for evaluation.

#### 2.2.5. Analysis of CPs and CAs

Chemical analyses were performed as previously described [5], with some modifications. The wood samples were humidified with water and then placed in a sealed glass container at 50 °C (around 0.2 mL water/5 g of sample in 100 mL air volume) and sampled for one hour. Chemicals emitted in the air were adsorbed by solid-phase micro-extraction (SPME) onto an 85 µm polyacrylate-fused silica fiber (Supelco, article number 57304) and desorbed and analyzed with gas-chromatography-mass spectrometry (GC-MS) using an Agilent Technologies GC (model 7890A, Santa Clara, CA, USA), a 60-m Zebron ZB-5 column (0.32 mm i.d., 1 µm film thickness, Phenomenex ApS, Værløse, Denmark), helium as carrier gas (purity 6.0, AGA Gas AB), and an oven temperature increasing from 35 to 290 °C. The eluate entered a quadrupole mass spectrometer (model 7995C, Agilent Technologies), and the characteristic fragments of CAs and CPs were detected in scan mode using MSD productivity ChemStation software (Revision E.02.00 and E.02.02) and identified using a mass spectrum library (Wiley7N, Wiley, Hoboken, HJ, USA). The following substances were analyzed: PCP, pentaCA (PCA); 2,3,4,6-tetraCP; 2,3,4,5-tetraCP; 2,3,4,6-tetraCA; 2,3,5,6-tetraCA; 2,4,6-triCP; 2,4,6-triCA; and 2,4,5-triCP. Their relative amounts were calculated as fractions of the total ion chromatogram (TIC). The laboratory is accredited by the Swedish Board for Accreditation and Conformity Assessment, with accreditation number 2085.

### 2.3. Statistical Analyses

The relationship between the presence of the odor and the extent of the fungi was evaluated using the Kruskal–Wallis test at a significance level of 0.05 (Table 1).

## 3. Results

### 3.1. Litterature Search

The search on CPs/CAs and mold/mildew in the Web of Science (WoS) (see File S1) resulted in 34 hits [5,6,7,37,45,46,47,48,49,50,51,52,53,54,55,56,57,58,59,60,61,62,63,64,65,66,67,68,69,70,71,72,73,74], in sharp contrast with the 22,215 hits on CPs or CAs (not restricted to mold/mildew). Most of the 34 papers address tainted wine and/or cork and technical aspects such as analytical methods. Seven studies deal with the indoor environment (including three dealing with museums/archives), three of which originated from our group [5,6,7] and one from a German group [37]. None of the 34 studies addresses odor in relation to the extent of mold on treated wood. One study [55] describes visible mold in wine cellars but not on treated wood.

The same search string in PubMed (see File S1) yielded 542 hits in contrast to 11,556 hits before restricting it to mold/mildew. Of these 542 papers, 28 remained [5,6,15,16,37,52,55,65,75,76,77,78,79,80,81,82,83,84,85,86,87,88,89,90,91,92,93,94] after excluding studies based on the title. Based on scrutiny of full papers or abstracts, none of the 28 studies addresses odor from PCs/CAs in relation to the extent of mold on treated wood. Same as in the WoS search, one study [55] describes visible mold in wine cellars but not on CP-treated wood.

### 3.2. Author’s Investigation of Two Schools with Odor Problems

Two schools built in the 1960s were investigated; both had odor problems even though attempts had been made to remediate odor by under-pressurizing the crawlspaces by blocking air ventilation openings in the crawlspaces and pumping crawlspace air to the outside. In 2015, complaints emerged in school A about a “strange” odor. This was investigated in 2016 by a major company [95], which reported a deviant smell of a chemical nature in craft halls, entrances, and the girls’ changing rooms (other rooms were not examined). The odor came from the crawl space where rot-protected building parts and microbial growth were confirmed by visual inspection, laboratory analyses of CPs, CAs, and microscopy [95]. In school B, odor and health symptoms had been a problem for many years, according to a survey by the Urban Property Department of the City of Gothenburg. Six out of six of the staff members reported problems with different types of odors constantly (4/6) or mostly in the mornings (2/6). They used the odor descriptors moldy (5/6) or musty (1/6). Most of the staff reported health symptoms (5/6), such as fatigue (4/6), headache (4/6), nausea (1/6), and dizziness (1/6). An investigation was performed in 2017 by a major company [96]. Among other circumstances, chemical analyses demonstrated CPs in the wooden crawlspace ceilings; no microbiological investigation was performed [96].

**Figure 2:**
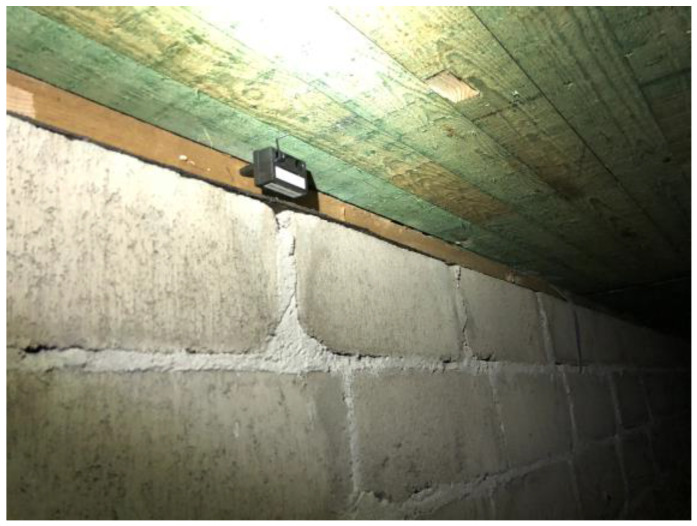
Crawlspace interior. The picture shows the concrete block wall, the treated wood sill plate on the wall (brown/green), and the treated wood ceiling (light green). A black humidity logger is placed on the sill plate, and two rectangular sampling sites are visible, one on the sill plate (far left) and one on the ceiling (middle).

When we investigated the two crawlspaces, both looked in good condition, but they had a distinct smell. Figure 2 shows an interior section of the crawlspace below school B. Limited and preliminary results from this school focused on technical aspects and, without chemical analyses, have been published as a conference paper [97].

All wood was treated in the crawlspaces below schools A and B, and there was no wood rot. Furthermore, no visual mold growth was found, with the possible exception in school A of some areas in the treated ceilings with a patchy thin white substance that could be mold but more likely was salt residue from preservatives [98]. Seven samples of treated wood were taken from school A and eight from school B. Immediately after sampling (sill plates and ceilings from different locations in the crawlspaces), several samples had the same odor as the crawlspace. Some samples also had a distinct odor when later evaluated in laboratories (Table 1). No visible mold was found on the samples; many looked like new and fresh pieces of treated wood. Still, all samples had some level of mold growth by microscopical evaluation (MI > 0), but only 3 of 15 had heavy growth (MI = 4) (Table 1). Analysis of data for odor and microscopic fungi on 15 sill plate samples (see Table 1) showed a clear correlation (*p* = 0.005). All samples contained at least one congener of both CP and CA, representing up to 50% of all detected volatiles in the chemical analyses.

## 4. Discussion

Our literature searches in the Web of Science and PubMed yielded zero results on studies that address odor in relation to the extent of mold on wood treated with chlorophenols. However, one German study from 2004 stated, without further details, that an increasing number of odorous houses had no indications of elevated levels of mold growth [37].

Thus, our experimental investigation in two schools with odor problems is the first to answer the question to what extent CP-treated wood contains mold. We show that none of the 15 treated wood samples from odorous crawlspaces from the 1960s contained mold when seen by the naked eye, but they did indeed contain varying amounts of mold when examined in a microscope. The amount of microscopic mold correlated with the odor. These results on microscopic amounts of mold have important implications for the hazard perception that evolved in relation to Swedish “mold buildings”.

A limitation of our study is that the number of samples is small. Still, the generalizability of our results can be evaluated by comparing the with two Swedish grey literature investigations that addressed our present questions [99,100]. They cover an additional 65 samples: 39 samples in a 1994 report from The Swedish Wood Preservation Institute, “Odor from impregnated wood” [99] and 26 samples in a 2010 exam work from the KTH Royal Institute of Technology, “Construction deficiencies in a terrace house area. Suggestions of reconstruction solutions” [100]. File S2 contains relevant information from these investigations, which we extracted, processed, and presented in English together with experts familiar with or involved in the investigations, i.e., Folke Björk (co-author) and Jöran Jermer (Acknowledgements).

Our result that treated wood contains mold when seen through a microscope but not by the naked eye is in line with the two studies [99,100]. Our finding is not surprising, as it has never been claimed that CPs can be used for sterilization, and numerous articles describe the microbial metabolism of CPs by certain microorganisms, not only methylation but also, for example, dechlorination, which leads to less toxic derivatives [101,102]. Microbial activity is taken advantage of in the bioremediation of contaminated soils and other media. Herein, we use the word mold as we analyzed filamentous fungi regardless of species. It is outside the scope of this article to investigate the species identity of fungi and even smaller microorganisms (prokaryotes, including Actinomycetes) that may grow on CP-treated wood, but we expect that the species pattern would vary depending on many factors, including the CP congener profile and the potential presence of other biocides. For example, the Swedish KP-Cuprinol also contained copper (in addition to CPs).

Our finding that the amount of microscopic mold on treated wood correlates with odor was not found in the two grey literature studies [99,100]. In our study, analyses of CAs and CPs were also performed on individual samples, and there may be a correlation with the amounts of CAs in the treated wood. Even though these findings may seem interesting to follow up in further studies, it is, in practice, difficult to standardize the different field procedures involved. Even if uniform samples could be extracted from constructions (size and form) and sent for analysis of microorganisms and chemicals, the results could differ depending on which kind of treatments had been made, for example, deep impregnation with KP-Cuprinol or BP-Hylosan or superficial treatment with other preservatives. Furthermore, results would likely differ substantially depending on when and where the samples were extracted. This is because moisture conditions may vary both in time and place. For example, treated wall sills or sill plates on a concrete structure may be moist only on some of its surfaces and/or at some times of the year or on certain occasions. Thus, it is, in practice, difficult to know where the microbial growth and biotransformation to CAs will occur. Therefore, in praxis, remedial action in odorous Swedish buildings often strives to remove all CP-treated wood, although physical and economic constraints may have to be considered. The praxis evolved a long time ago; it was reported in 1977 that “*fungi that attack impregnated wood often cause severe odor*” [103] and later that removal of impregnated wall sills could resolve the smell of mold, satisfy tenants, and combat “Sickhouses” [104].

Our starting point for this study was that Swedish impregnated wood played a key role in forming the perception of hazardous mold. Our main result is that such wood only contains microscopic levels of mold. On the one hand, the inconspicuous amount of mold is surprising as it contradicts the term “mold buildings”. On the other hand, the inconspicuous amount of mold is fully in line with reports from the SP Technical Research Institute of Sweden stating that visible mold was hard to find, even when opening constructions [41,42] (see reference [6] for a translated description of the odorous buildings). The terms “mold buildings” [40] and “mold houses” [105,106] were misleading, as mold became a suspected cause of increasing hypersensitivity and allergy [107], and the terms spread in the Swedish public health domain.

Meanwhile, Swedish investigators representing supervisory government agencies did not present any results on mold as they became influential at the international level. For example, no data on mold were provided when “mold buildings” with obnoxious odor [40] and “sick buildings” with mold infestation [108] were introduced at conferences by Tomas Lindvall and co-workers. Lindvall later co-founded the *Indoor Air* journal [7].

Our findings herein are fully in line with a statement of Jan Sundell, another former agency representative and long-time chief editor of *Indoor Air* [7]. When describing the last 50 years of indoor air science and reflecting on mold, Sundell stated that, other than odor, his own studies found no causative microbial agents that could explain associations with asthma and allergy [32].

Today, the odorous CAs are well recognized in Sweden, for example, when mold odor is discussed in an epidemiologic cohort study reporting allergic outcomes from birth to adolescence [109]. The key question that remains is what kind of hazard the mold odor indicated in this and many other epidemiologic studies. Concerning health effects, the microscopic mold found on treated wood surfaces seems of little consequence compared with the pesticides. As a prospect for further research, we are presently compiling data on the chemical exposure that occurred in the 1970s–1980s, around the time CPs were restricted and banned in many countries. At that time, residents may have been significantly exposed by several routes (inhalation, skin contact, hand-to-mouth). Today, concentrations of CPs and CAs in Swedish buildings are very low and unlikely to be detrimental to health per se, although the CAs may still cause unpleasant odors [5,6].

For the future, we propose that research should follow standard procedures for exposure assessment and toxicology, which is common practice in the work and general environment. Furthermore, ambiguous and misleading terms such as “mold houses”, “sick buildings”, “sick building syndrome”, and “dampness and mold” should be avoided, as they tend to neglect or obscure more important hazardous exposures, such as preservatives, fungicides, and pesticides.

## 5. Conclusions

Wood treated with toxic CPs, leading to the formation of odorous CAs, played a key role when Sweden became a leader in indoor air research and the perception of hazardous mold was formed.

We demonstrate for the first time in the health science domain that such wood is not moldy as seen by the naked eye. Yet, it is not sterile, as varying amounts of mold can be seen through a microscope. Moreover, our data suggest that perceived odor correlates with the amount of microscopic mold.

We conclude that superficial microscopic mold suffices to result in the biotransformation of CPs to CAs in quantities that cause odor. The limited amount of mold on treated wood provides further strength to the argument that the adverse health effects linked to mold odor may have been caused by the CPs.

## Figures and Tables

**Table 1:** Evaluation of odor and mold on 15 CP-treated wood samples. Samples (1–15) from crawl space sill plates (SP) and ceilings (C) from two schools (A and B) built in the 1960s were evaluated for mold (mold index 0–4), presence of chlorophenols (CPs) and chloroanisoles (CAs), and perceived odor as evaluated at laboratories in Lund (one person) and later in Uppsala (two persons).

School Sample	Sample Location	Odor			Mold Index	CPs and CAs Present in Samples
		Lund	Uppsala1	Uppsala2		
A 1	SP	No	No	No	2	Yes
A 2	SP	No	No	No	2	Yes
A 3	C	Yes	No	No	3	Yes ^1^
A 4	SP	No	No	No	2	Yes
A 5	SP	No	No	No	3	Yes
A 6	C	Yes	Yes	Yes	4	Yes ^1^
A 7	C	Yes	No	Yes	4	Yes ^1^
A 8	SP	No	No	No	2	Yes
B 9	SP	No	No	No	3	Yes
B 10	SP	No	Yes	No	3	Yes
B 11	C	No	Yes	No	4	Yes ^1^
B 12	C	No	No	No	1	Yes
B 13	SP	No	No	No	2	Yes
B 14	SP	No	No	No	2	Yes
B 15	C	No	No	No	1	Yes

^1^ Samples where individual congeners represented 5% or more of all volatiles.

## Data Availability

The data used to support the findings of this study are included within the article.

## Supplementary Materials

The following supporting information can be downloaded at: https://www.mdpi.com/article/10.3390/microorganisms12020395/s1, File S1: Literature search on relations between chlorophenols, chloroanisoles and mold, Table S1: Chlorophenol (CP) AND chloroanisole (CA) congeners and their Chemical Abstract Service Registry (CAS) numbers. File S2: Previous Swedish investigations not reported in the scientific domain on relations between odor and mold growth on impregnated wood. Table S2: Evaluation of odor and micro-fungi on 39 CP-treated wood samples. Table S3: Evaluation of odor and microorganisms on 26 CP-treated wood samples.
