# Supplementary material for: Mold Odor from Wood Treated with Chlorophenols despite Mold Growth That Can Only Be Seen Using a Microscope
Source: Microorganisms. 2024 Feb 16;12(2):395. doi: 10.3390/microorganisms12020395 (PMC10891868; doi:10.3390/microorganisms12020395)
Supplement: Supplementary file 1 [file microorganisms-12-00395-s001.zip › microorganisms-2858342 supplementary/File S1.pdf]

Mold Odor from Wood Treated with Chlorophenols Despite Mold Growth That Can Only Be Seen Using a Microscope

Johnny C. Lorentzen, Olle Ekberg, Maria Alm, Folke Björk, Lars-Erik Harderup, and Gunnar Johanson

File S1. Literature search on relations between chlorophenols, chloroanisoles and mold

Web of Science and PubMed were searched using the search string: "(mold OR mould OR mildew) AND (chlorophenol: OR monochlorophenol: OR dichlorophenol: OR trichlorophenol: OR tetrachlorophenol: OR pentachlorophenol: OR chloroanisole: OR monochloroanisole: OR dichloroanisole: OR trichloroanisole: OR tetrachloroanisole: OR pentachloroanisole:)". In addition to the chemical names, all relevant CAS numbers (Table S1) were included in the search. The search was performed on 16 October 2023.

Table S1. Chlorophenol (CP) AND chloroanisole (CA) congeners and their Chemical Abstract Service Registry (CAS) numbers.

Chlorophenol	CAS no.	Corresponding chloroanisole	CAS no.
Monochlorophenols			
CP	25167-80-0		
2-CP	95-57-8	2-CA	766-51-8
3-CP	108-43-0	3-CA	2845-89-8
2-CP or 3-CP	29353-84-2		
4-CP	106-48-9	4-CA	623-12-1
Dichlorophenols			
diCP	25167-81-1		
2,3-diCP	576-24-9	23-diCA	1984-59-4
2,3-diCP or 2,4-diCP	83700-00-9		
2,4-diCP	120-83-2	2,4-diCA	533-82-2
2,5-diCP	583-78-8	2,5-diCA	1984-58-3
2,6-diCP	87-65-0	2,6-diCA	1984-65-2
3,4-diCP	95-77-2	3,4-diCA	36404-30-5
3,5-diCP	591-35-5	3,5-diCA	33719-74-3
Trichlorophenols			
triCP	25167-82-2		
2,3,4-triCP	15950-66-0	2,3,4-triCA	54135-80-7
2,3,5-triCP	933-78-8	2,3,5-triCA	54135-81-8
2,3,6-triCP	933-75-5	2,3,6-triCA	50375-10-5
2,4,5-triCP	95-95-4	2,4,5-triCA	6130-75-2
2,4,6-triCP	88-06-2	2,4,6-triCA	87-40-1
2,4,?-triCP 1	95719-03-2		
3,4,5-triCP	609-19-8	3,4,5-triCA	609-19-8

Chlorophenol	CAS no.	Corresponding chloroanisole	CAS no.
Tetrachlorophenols			
tetraCP	25167-83-3		
2,3,4,5-tetraCP	4901-51-3	2,3,4,5-tetraCA	938-86-3
2,3,4,6-tetraCP	58-90-2	2,3,4,6-tetraCA	938-22-7
2,3,5,6-tetraCP	935-95-5	2,3,5,6-tetraCA	6936-40-9
2,4,?,?-tetraCP 1	95719-04-3	2,3,5,6-tetraCA	6936-40-9
Pentachlorophenol			
PCP	87-86-5	PCA	1825-21-4

[1] ? means unspecified position of the carbon.

Results

Web of Science

The search in Web of Science resulted in 34 hits, listed below. None of the 34 studies addresses odor from PCs/CAs in relation to the extent of mold on treated wood. One study [12] describes visible mold in wine cellars, but not on CP-treated wood. Four of the papers [11,17-19] are cited in our manuscript for other reasons.

1. Bliffeld, M.; Mundy, J.; Potrykus, I.; Fütterer, J. Genetic engineering of wheat for increased resistance to powdery mildew disease. Theor. Appl. Genet. 1999, 98, 1079-1086, doi:10.1007/s001220051170.
2. Chen, J.B.; Ma, Y.Y.; Lin, H.P.; Zheng, Q.Z.; Zhang, X.X.; Yang, W.B.; Li, R. Fabrication of Hydrophobic ZnO/PMHS Coatings on Bamboo Surfaces: The Synergistic Effect of ZnO and PMHS on Anti-Mildew Properties. Coatings 2019, 9, 10, doi:10.3390/coatings9010015.
3. Corsi, A.J.; Hernandez, F.C.R.; Cruz, G.C.Y.; Neal, J.A. The effectiveness of electron beam irradiation to reduce or eliminate mould in cork stoppers. Int. J. Food Sci. Technol. 2016, 51, 389-395, doi:10.1111/ijfs.12993.
4. Dang, X.J.; Stevenson, K.J.; Hupp, J.T. Monitoring molecular adsorption on high-area titanium dioxide via modulated diffraction of visible light. Langmuir 2001, 17, 3109-3112, doi:10.1021/la010096q.
5. Deering, K.; Spiegel, E.; Quaisser, C.; Nowak, D.; Rakete, S.; Garí, M.; Bose-O'Reilly, S. Exposure assessment of toxic metals and organochlorine pesticides among employees of a natural history museum. Environ. Res. 2020, 184, 11, doi:10.1016/j.envres.2020.109271.
6. Deering, K.; Spiegel, E.; Quaisser, C.; Nowak, D.; Schierl, R.; Bose-O'Reilly, S.; Garí, M. Monitoring of arsenic, mercury and organic pesticides in particulate matter, ambient air and settled dust in natural history collections taking the example of the Museum fur Naturkunde, Berlin. Environ. Monit. Assess. 2019, 191, 17, doi:10.1007/s10661-019-7495-z.
7. deJong, E.; Field, J.A. Sulfur tuft and turkey tail: Biosynthesis and biodegradation of organohalogens by basidiomycetes. Annu. Rev. Microbiol. 1997, 51, 375-414, doi:10.1146/annurev.micro.51.1.375.
8. Endo, M.; Matsui, C.; Maeta, N.; Uehara, Y.; Matsuda, R.; Fujii, Y.; Fujita, A.; Fujii, T.; Yamada, O. Growth characteristics of Aspergillus oryzae in the presence of 2,4,6-trichlorophenol. J. Gen. Appl. Microbiol. 2021, 67, 256-259, doi:10.2323/jgam.2021.06.001.
9. Gabrielli, M.; Englezos, V.; Rolle, L.; Segade, S.R.; Giacosa, S.; Cocolin, L.; Paissoni, M.A.; Lambri, M.; Rantsiou, K.; Maury, C. Chloroanisoles occurrence in wine from grapes subjected to electrolyzed water treatments in the vineyard. Food Res. Int. 2020, 137, 8, doi:10.1016/j.foodres.2020.109704.
10. Giacosa, S.; Gabrielli, M.; Torchio, F.; Segade, S.R.; Grobas, A.M.M.; Aimonino, D.R.; Gay, P.; Gerbi, V.; Maury, C.; Rolle, L. Relationships among electrolyzed water postharvest treatments on winegrapes and chloroanisoles occurrence in wine. Food Res. Int. 2019, 120, 235-243, doi:10.1016/j.foodres.2019.02.034.
11. Gunschera, J.; Fuhrmann, F.; Salthammer, T.; Schulze, A.; Uhde, E. Formation and emission of chloroanisoles as indoor pollutants. Environ. Sci. Pollut. Res. 2004, 11, 147-151, doi:10.1007/bf02979668.

12. Haas, D.; Galler, H.; Habib, J.; Melkes, A.; Schlacher, R.; Buzina, W.; Friedl, H.; Marth, E.; Reinthaler, F.F. Concentrations of viable airborne fungal spores and trichloroanisole in wine cellars. Int. J. Food Microbiol. 2010, 144, 126-132, doi:10.1016/j.ijfoodmicro.2010.09.008.
13. Henry, C. NMR method detects spoiled wine in unopened bottles. Chem. Eng. News 2005, 83, 34-35.
14. Kim, C.M.; Ullah, A.; Kim, K.G.; Kim, S.Y.; Kim, G.M. Preparation of Carbon Nanotube-Wrapped Porous Microparticles Using a Microfluidic Device. J. Nanosci. Nanotechnol. 2016, 16, 12003-12008, doi:10.1166/jnn.2016.13633.
15. Lehtaru, J. Preservation of Archival Records at the Estonian National Archives through the Century. Part 2. Tuna-Ajalookultuuri Ajak. 2021, 82-+.
16. Li, Y.Q.; Li, W.C.; Wang, Y.H.; Zhou, H.L.; Hu, G.J.; Zhang, N.H.; Sun, C. Development of a solid-phase microextraction fiber coated with poly(methacrylic acid-ethylene glycol dimethacrylate) and its application for the determination of chlorophenols in water coupled with GC. J. Sep. Sci. 2013, 36, 2121-2127, doi:10.1002/jssc.201200979.
17. Lorentzen, J.C.; Harderup, L.E.; Johanson, G. Evidence of Unrecognized Indoor Exposure to Toxic Chlorophenols and Odorous Chloroanisoles in Denmark, Finland, and Norway. Indoor Air 2023, 2023, 9, doi:10.1155/2023/2585089.
18. Lorentzen, J.C.; Juran, S.A.; Ernstgård, L.; Olsson, M.J.; Johanson, G. Chloroanisoles and Chlorophenols Explain Mold Odor but Their Impact on the Swedish Population Is Attributed to Dampness and Mold. Int. J. Environ. Res. Public Health 2020, 17, 20, doi:10.3390/ijerph17030930.
19. Lorentzen, J.C.; Juran, S.A.; Nilsson, M.; Nordin, S.; Johanson, G. Chloroanisoles may explain mold odor and represent a major indoor environment problem in Sweden. Indoor Air 2016, 26, 207-218, doi:10.1111/ina.12207.
20. Muthusubramanian, L.; Mitra, R.B. A new approach to the synthesis of bromochloromethane as a biocide intermediate. J. Soc. Leather Technol. Chem. 2005, 89, 34-35.
21. Pereira, C.S.; Marques, J.J.F.; San Romao, M.V. Cork taint in wine: Scientific knowledge and public perception - A critical review. Crit. Rev. Microbiol. 2000, 26, 147-162, doi:10.1080/10408410008984174.
22. Pereira, C.S.; Pires, A.; Valle, M.J.; Boas, L.V.; Marques, J.J.F.; San Romao, M.V. Role of Chrysonilia sitophila in the quality of cork stoppers for sealing wine bottles. J. Ind. Microbiol. Biotechnol. 2000, 24, 256-261, doi:10.1038/sj.jim.2900815.
23. Philipp, C.; Sari, S.; Brandes, W.; Nauer, S.; Patzl-Fischerleitner, E.; Eder, R. Reduction in Off-Flavors in Wine Using Special Filter Layers with Integrated Zeolites and the Effect on the Volatile Profile of Austrian Wines. Appl. Sci.-Basel 2022, 12, 18, doi:10.3390/app12094343.
24. Prak, S.; Gunata, Z.; Guiraud, J.P.; Schorr-Galindo, S. Fungal strains isolated from cork stoppers and the formation of 2,4,6-trichloroanisole involved in the cork taint of wine. Food Microbiol. 2007, 24, 271-280, doi:10.1016/j.fm.2006.05.002.
25. Prohl, A.; Boge, K.P.; AlsenHinrichs, C. Activities of an Environmental Analysis Van in the German Federal State Schleswig-Holstein. Environ. Health Perspect. 1997, 105, 844-849, doi:10.2307/3433702.
26. Rocha, S.; Delgadillo, I.; Correia, A.J.F. GC-MS study of volatiles of normal and microbiologically attacked cork from Quercus suber L. J. Agric. Food Chem. 1996, 44, 865-871, doi:10.1021/jf9500400.
27. Schnürer, J.; Olsson, J.; Börjesson, T. Fungal volatiles as indicators of food and feeds spoilage. Fungal Genet. Biol. 1999, 27, 209-217, doi:10.1006/fgbi.1999.1139.
28. Shehu, R.A.; Al-Hamidi, A.A.A.; Rabbani, N.; Duhaiman, A.S. Inhibition of camel lens ζ-crystallin/NADPH:Quinone oxidoreductase activity by chlorophenols. J. Enzym. Inhib. 1998, 13, 229-236, doi:10.3109/14756369809028343.
29. Uraki, Y.; Kubo, S.; Sano, Y. Preparation of activated carbon moldings from the mixture of waste newspaper and isolated lignins: mechanical strength of thin sheet and adsorption property. J. Wood Sci. 2002, 48, 521-526, doi:10.1007/bf00766650.
30. Varelas, V.; Sanvicens, N.; Marco, M.P.; Kintzios, S. Development of a cellular biosensor for the detection of 2,4,6-trichloroanisole (TCA). Talanta 2011, 84, 936-940, doi:10.1016/j.talanta.2011.02.029.
31. Vlachos, P.; Kampioti, A.; Kornaros, M.; Lyberatos, G. Development and evaluation of alternative processes for sterilization and deodorization of cork barks and natural cork stoppers. Eur. Food Res. Technol. 2007, 225, 653-663, doi:10.1007/s00217-006-0461-3.

32. Wörle, M.; Hubert, V.; Hildbrand, E.; Hunger, K.; Lehmann, E.; Mayer, I.; Petrak, G.; Pracher, M.; von Arx, U.; Wülfert, S. Evaluation of decontamination methods of pesticide contaminated wooden objects in museum collections: Efficiency of the treatments and influence on the wooden structure. J. Cult. Herit. 2012, 13, S209-S215, doi:10.1016/j.culher.2012.01.006.
33. Yang, M.; Zheng, S.K. Pollutant removal-oriented yeast biomass production from high-organic-strength industrial wastewater: A review. Biomass Bioenerg. 2014, 64, 356-362, doi:10.1016/j.biombioe.2014.03.020.
34. Yapici, B.M.; Karaboz, I. The effect of two anti-fungal compounds on the growth of molds that frequently appear on tanned leather. J. Am. Leather Chem. Assoc. 1997, 92, 38-45.

PubMed

The search in PubMed resulted in 542 hits (not shown). Of these 542 papers, 28 remained (listed below) after excluding studies based on title. Based on full papers [8,9,16,17,21,23] or abstracts (remaining papers), none of the 28 studies addresses odor from PCs/CAs in relation to the extent of mold on treated wood. One study [9, same as 12 in the Web of Science search] describes visible mold in wine cellars, but not on CP-treated wood. Three studies [8,16,17] are cited in our manuscript for other reasons. Notably, only 12 of the 34 hits in Web of Science [5,6,8,11,12,16,18,19, 24,25,28,30] were found among the 542 hits in PubMed.

1. Alleman, B.C.; Logan, B.E.; Gilbertson, R.L. Toxicity of pentachlorophenol to six species of white rot fungi as a function of chemical dose. Applied and environmental microbiology 1992, 58, 4048-4050, doi:10.1128/aem.58.12.4048-4050.1992.
2. Beliakova, L.A. [Pentachlorophenolate sodium as an antiseptic preventing mold formation in the glue]. Mikrobiologiia 1956, 25, 713-717.
3. Cserjesi, A.J. The adaptation of fungi to pentachlorophenol and its biodegradation. Canadian journal of microbiology 1967, 13, 1243-1249, doi:10.1139/m67-169.
4. Cserjesi, A.J.; Johnson, E.L. Methylation of pentachlorophenol by Trichoderma virgatum. Canadian journal of microbiology 1972, 18, 45-49, doi:10.1139/m72-007.
5. Curtis, F.; Dennis, C.; Gee, J.M.; Gee, M.G.; Griffiths, N.M.; Land, D.G.; Peel, J.L.; Robinson, D. Chloroanisoles as a cause of musty taint in chickens and their microbiological formation from chlorophenols in broiler house litters. Journal of the science of food and agriculture 1974, 25, 811-828, doi:10.1002/jsfa.2740250711.
6. Duncan, C.G.; Deverall, F.J. Degradation of Wood Preservatives by Fungi. Applied microbiology 1964, 12, 57-62, doi:10.1128/am.12.1.57-62.1964.
7. Endo, M.; Matsui, C.; Maeta, N.; Uehara, Y.; Matsuda, R.; Fujii, Y.; Fujita, A.; Fujii, T.; Yamada, O. Growth characteristics of Aspergillus oryzae in the presence of 2,4,6-trichlorophenol. The Journal of general and applied microbiology 2021, 67, 256-259, doi:10.2323/jgam.2021.06.001.
8. Gunschera, J.; Fuhrmann, F.; Salthammer, T.; Schulze, A.; Uhde, E. Formation and emission of chloroanisoles as indoor pollutants. Environmental science and pollution research international 2004, 11, 147-151, doi:10.1007/bf02979668.
9. Haas, D.; Galler, H.; Habib, J.; Melkes, A.; Schlacher, R.; Buzina, W.; Friedl, H.; Marth, E.; Reinthaler, F.F. Concentrations of viable airborne fungal spores and trichloroanisole in wine cellars. International journal of food microbiology 2010, 144, 126-132, doi:10.1016/j.ijfoodmicro.2010.09.008.
10. Higson, F.K. Degradation of xenobiotics by white rot fungi. Reviews of environmental contamination and toxicology 1991, 122, 111-152, doi:10.1007/978-1-4612-3198-1_4.
11. Hofrichter, M.; Bublitz, F.; Fritsche, W. Unspecific degradation of halogenated phenols by the soil fungus Penicillium frequentans Bi 7/2. Journal of basic microbiology 1994, 34, 163-172, doi:10.1002/jobm.3620340306.
12. Komorowicz, M.; Janiszewska-Latterini, D.; Przybylska-Balcerek, A.; Stuper-Szablewska, K. Fungal Biotransformation of Hazardous Organic Compounds in Wood Waste. Molecules (Basel, Switzerland) 2023, 28, doi:10.3390/molecules28124823.
13. Kremer, S.; Sterner, O.; Anke, H. Degradation of pentachlorophenol by Mycena avenacea TA 8480--identification of initial dechlorinated metabolites. Zeitschrift fur Naturforschung C, Journal of biosciences 1992, 47, 561-566, doi:10.1515/znc-1992-7-812.

14. Lamar, R.T.; Dietrich, D.M. In Situ Depletion of Pentachlorophenol from Contaminated Soil by Phanerochaete spp. Applied and environmental microbiology 1990, 56, 3093-3100, doi:10.1128/aem.56.10.3093-3100.1990.
15. Leontievsky, A.A.; Myasoedova, N.M.; Baskunov, B.P.; Evans, C.S.; Golovleva, L.A. Transformation of 2,4,6-trichlorophenol by the white rot fungi Panus tigrinus and Coriolus versicolor. Biodegradation 2000, 11, 331-340, doi:10.1023/a:1011154209569.
16. Lorentzen, J.C.; Juran, S.A.; Ernstgård, L.; Olsson, M.J.; Johanson, G. Chloroanisoles and Chlorophenols Explain Mold Odor but Their Impact on the Swedish Population Is Attributed to Dampness and Mold. International journal of environmental research and public health 2020, 17, doi:10.3390/ijerph17030930.
17. Lorentzen, J.C.; Juran, S.A.; Nilsson, M.; Nordin, S.; Johanson, G. Chloroanisoles may explain mold odor and represent a major indoor environment problem in Sweden. Indoor air 2016, 26, 207-218, doi:10.1111/ina.12207.
18. Mileski, G.J.; Bumpus, J.A.; Jurek, M.A.; Aust, S.D. Biodegradation of pentachlorophenol by the white rot fungus Phanerochaete chrysosporium. Applied and environmental microbiology 1988, 54, 2885-2889, doi:10.1128/aem.54.12.2885-2889.1988.
19. Montiel-González, A.M.; Fernández, F.J.; Keer, N.; Tomasini, A. Increased PCP removal by Amylomyces rouxii transformants with heterologous Phanerochaete chrysosporium peroxidases supplementing their natural degradative pathway. Applied microbiology and biotechnology 2009, 84, 335-340, doi:10.1007/s00253-009-1981-0.
20. Pezzotti, F.; Okrasa, K.; Therisod, M. Oxidation of chlorophenols catalyzed by Coprinus cinereus peroxidase with in situ production of hydrogen peroxide. Biotechnology progress 2004, 20, 1868-1871, doi:10.1021/bp049750t.
21. Pröhl, A.; Böge, K.P.; Alsen-Hinrichs, C. Activities of an Environmental Analysis Van in the German Federal State Schleswig-Holstein. Environmental health perspectives 1997, 105, 844-849, doi:10.1289/ehp.105-1470208.
22. Reddy, G.V.B.; Gold, M.H. Degradation of pentachlorophenol by Phanerochaete chrysosporium: intermediates and reactions involved. Microbiology 2000, 146 (Pt 2), 405-413, doi:10.1099/00221287-146-2-405.
23. Rose, L.J.; Simmons, R.B.; Crow, S.A.; Ahearn, D.G. Volatile organic compounds associated with microbial growth in automobile air conditioning systems. Current microbiology 2000, 41, 206-209, doi:10.1007/s002840010120.
24. Schmidhalter, D.R.; Canevascini, G. Isolation and characterization of the cellobiose dehydrogenase from the brown-rot fungus Coniophora puteana (Schum ex Fr.) Karst. Archives of biochemistry and biophysics 1993, 300, 559-563, doi:10.1006/abbi.1993.1077.
25. Seigle-Murandi, F.; Steiman, R.; Benoit-Guyod, J.L. Biodegradation potential of some micromycetes for pentachlorophenol. Ecotoxicology and environmental safety 1991, 21, 290-300, doi:10.1016/0147-6513(91)90068-z.
26. Shirk, H.G.; Poelma, P.L.; Corey, R.R., Jr. The influence of chemical structure on fungal activity. I. Effect of p-chlorophenol and derivatives. Archives of biochemistry and biophysics 1951, 32, 386-391, doi:10.1016/0003-9861(51)90287-1.
27. Tayal, A.K.; Das, L.; Kaur, I. Biodegradation of pentachlorophenol (PCP) by white rot fungal strains screened from local sources and its estimation by high-performance liquid chromatography. Biomedical chromatography : BMC 1999, 13, 220-224,doi:10.1002/(sici)1099-0801(199905)13:3<220::Aid-bmc823>3.0.Co;2-e.
28. Tortella, G.R.; Diez, M.C.; Duran, N. Fungal diversity and use in decomposition of environmental pollutants. Critical reviews in microbiology 2005, 31, 197-212, doi:10.1080/10408410500304066.
