# Supplementary material for: Mold Odor from Wood Treated with Chlorophenols despite Mold Growth That Can Only Be Seen Using a Microscope
Source: Microorganisms. 2024 Feb 16;12(2):395. doi: 10.3390/microorganisms12020395 (PMC10891868; doi:10.3390/microorganisms12020395)
Supplement: Supplementary file 1 [file microorganisms-12-00395-s001.zip › microorganisms-2858342 supplementary/File S2.pdf]

Mold Odor from Wood Treated with Chlorophenols Despite Mold Growth That Can Only Be Seen Using a Microscope

Johnny C. Lorentzen, Olle Ekberg, Maria Alm, Folke Björk, Lars-Erik Harderup, and Gunnar Johanson

File S2. Previous Swedish investigations not reported in the scientific domain on relations between odor and mold growth on impregnated wood.

We identified two previous investigations of relevance for our study questions. The first is from 1994, a report from The Swedish Wood Preservation Institute: "Odor from impregnated wood" [1]. The second is from 2010, an exam work from the KTH Royal Institute of Technology: "Construction deficiencies in a terrace house area. Suggestions of reconstruction solutions" [2].

The Swedish Wood Preservation Institute, 1994

This report [1] ascertains that *"Nasty odor in houses is a problem that has attracted attention especially in houses built in the 1960s and 70s, when constructive moisture protection was of secondary importance. The nasty smell was associated early on with especially the impregnated timber in the foundation structure, and many characterized it as mold smell and the houses analogously as mold houses"* (our translation). The odor only develops in wood impregnated with preservatives containing CPs. Swedish KP-Cuprinol is specifically mentioned. The report describes a high prevalence of self-reported asthma and other health effects in an area with many "mold houses" [1]. Analysis of around 30 cases where abnormal moisture load occurred on impregnated sill plates showed that the presence of mold was usually not as remarkable as the smell. The smell became, due to its remarkably "nasty" character, dimensioning for the measures, which was often sill replacement. Our analysis of data for odor and microscopic mold (the authors use the word micro-fungi) on 39 sill plate samples, see Table B1, showed no correlation ($p=0.36$, as evaluated with the Kruskal Wallis test at a significance level of 0.05).

Table S2. Evaluation of odor and micro-fungi on 39 CP-treated wood samples.

Odor	Presence of micro-fungi using a microscope		
	Scarce	Middle	Abundant
Nasty	12	6	5
Normal	11	3	2

KTH Royal Institute of Technology, 2010

This exam work [2] recommends replacement of all sill plates in an area in Uppsala with around 240 apartments where indoor environment investigations were common. The sill plates were not shown to be treated with CPs, but this was presupposed. The study contains a section on KP-Cuprinol and references the report from the Wood Preservative Institute described above, which states that KP-Cuprinol is responsible for odor in "moldy buildings" [2]. Moreover, an air sampling result from an apartment in the area identified 2,4,6-triCA, according to an investigation obtained from the landlord, Uppsalahem AB (performed by the company Bjerking, their reference no. 29178, dated 23 June 2009). This is a clear

indication that the sill plates indeed contained CPs. The investigation describes the random selection and investigation of 14 apartments [2]. In each apartment, two openings were made in walls, facing south and north, 28 holes in total. Three wood samples were taken from each hole: sill plate; joist next to the sill plate; and joist 1.8 meter from the sill plate; altogether 84 samples. Deviant smell before opening the walls was observed at three of the 28 locations (11 %). In each hole, all sill plates were visibly treated with a preservative. When taking wood samples, 25/28 of sampled sill plates had "odor of impregnation", 4/28 of adjacent joist samples had deviant odor, and 0/28 joist samples at 1.8-meter distance had deviant odor. Mold was visible on 1/84 of sill plate samples, 6/84 of adjacent joist samples and 6/84 of joist samples at 1.8-meters distance. Using a microscope, mold (the authors use the word microorganisms) was detected on 19/84 of sill plate samples, 18/84 of adjacent joist samples and 8/84 of joist samples at 1.8 meters distance. The report contains data on moisture ratio in the wood samples (not shown here): highest in sill plates; middle for adjacent joists; and lowest for joists at 1.8-meters distance. There was a clear association between moisture ratio and microscopic mold. A panel of 10 persons categorized the odor from the 84 samples (840 tests) as "distinct", "weak", or "none". Statistical analyses showed that pronounced odor increased the probability of encountering impregnated wood around 20 times (p=0.003). There was no association between microscopic mold and odor or sample type [2].

Beside these data, and using raw data attached to the investigation [2], we compiled results for individual sill plate samples, excluding two samples that had not been fully evaluated for odor, yielding 26 samples of treated wood. Analysis of data for odor score and presence of microscopic mold on 26 sill plate samples (Table B2) showed no significant correlation, albeit a trend (p=0.07, as evaluated with the Kruskal Wallis test at a significance level of 0.05).

Table S3. Evaluation of odor and microorganisms on 26 CP-treated wood samples.

Sample[1]	Odor score				Microorganisms	
	Distinct	Weak	None	Total score	Microscopic	Macroscopic
	(Number of odor panelists)			(range 0-20)		
1, S	5	4	1	14	Yes	No
1, N	9	1	0	19	Yes	No
2, S	0	8	2	8	No	No
2, N	8	2	0	18	Yes	No
3, S	6	3	1	15	Yes	No
3, N	3	6	1	12	Yes	No
4, S	3	7	0	13	No	No
4, N	1	7	2	9	Yes	No
5, S	7	3	0	10	No	No
5, N	4	6	0	14	Yes	No
6, S	7	2	1	16	Yes	No
6, N	9	1	0	19	Yes	No
7, S	0	7	3	7	Yes	Yes
7, N	3	7	0	13	Yes	No
8, S	5	4	1	14	No	No
8, N	2	8	0	12	Yes	No
9, S	6	2	2	14	No	No
9, N	4	6	0	14	Yes	No
10, S	4	4	2	12	No	No

Sample[1]	Odor score				Microorganisms	
	Distinct	Weak	None	Total score	Microscopic	Macroscopic
	(Number of odor panelists)			(range 0-20)		
10, N	3	5	2	11	No	No
11, S	2	8	0	12	Yes	No
11, N	3	6	1	12	Yes	No
12, S	8	1	1	17	Yes	No
12, N	5	5	0	15	Yes	No
13, S	4	5	1	13	Yes	No
13, N	6	4	0	16	Yes	Yes

[1] S and N indicate that the samples were taken from walls facing south and north, respectively. This information enables the tracking of samples and data in the original investigation.

References

1. Nyman, E. Lukt från impregnerat trä (in Swedish); Svenska Träskyddsinstitutet: Stockholm, Sweden, 1994; pp. 1-29.
2 Norén, Y. Construction deficiencies in a terrace house area. Suggestions of reconstruction solutions (in Swedish with abstract in Eglish). M.Sc. thesis, KTH Royal Institute of Technology, Stockholm, Sweden, April 2010.
